# Repressors of mTORC1 act to blunt the anabolic response to feeding in the soleus muscle of a cast‐immobilized mouse hindlimb

**DOI:** 10.14814/phy2.13891

**Published:** 2018-10-18

**Authors:** Kevin L. Shimkus, Leonard S. Jefferson, Bradley S. Gordon, Scot R. Kimball

**Affiliations:** ^1^ Department of Cellular and Molecular Physiology The Pennsylvania State University College of Medicine Hershey Pennsylvania; ^2^ College of Human Sciences Florida State University Tallahassee Florida

**Keywords:** Anabolic resistance, casting, DEPTOR, disuse atrophy, REDD1, Sestrin1

## Abstract

We recently reported results showing that cast immobilization of a rat hindlimb rapidly leads to development of anabolic resistance as demonstrated by failure of oral leucine administration to activate the mechanistic target of rapamycin complex 1 (mTORC1) and stimulate protein synthesis in the soleus muscle. The goal of this study was to assess the possible contribution of several mTORC1 regulatory proteins to the development of anabolic resistance. To accomplish this, 14‐week‐old male C57BL/6 mice (*n* = 21) were subjected to unilateral cast immobilization of the hindlimb for either 1 or 3 days, and the immobilized limb was compared to its contralateral control. The mass of the soleus muscle was decreased in the immobilized compared to the non‐immobilized limb within 72‐h in association with diminished protein synthesis. In agreement with our previous report, a 24‐h casting period was sufficient to induce anabolic resistance, as demonstrated by blunted re‐feeding‐induced activation of mTORC1. Moreover, resistance of mTORC1 activation was associated not only with upregulated expression of REDD1, but also with altered expression of other mTORC1 regulatory proteins, that is, Sestrin1 and DEP domain‐containing mTOR interacting protein (DEPTOR). In addition, re‐feeding‐induced phosphorylation of DEPTOR was significantly impaired in the immobilized compared to the non‐immobilized limb. This work builds upon previous discoveries by our laboratory to elucidate the blunted mTORC1 response to stimuli during disuse of skeletal muscle induced by cast immobilization while highlighting new potential therapeutic targets for future countermeasures against muscle atrophy.

## Introduction

Loss of muscle mass and function is a universal response to disuse, for example, limb immobilization, extended bed rest, or space flight, that can lead not only to impaired quality of life but in some cases to increased morbidity and mortality (Fujita et al. 1995; Rantanen et al. 2000). In part, the loss of muscle mass during disuse is due to the development of anabolic resistance, a condition defined as a decrease in the response of muscle protein synthesis to an anabolic stimulus such as nutrients, specifically amino acids (Cuthbertson et al. 2005). Amino acids act to stimulate muscle protein synthesis by activating a protein kinase referred to as the mechanistic/mammalian target of rapamycin in complex 1 (mTORC1) (Anthony et al. 2000). Activated mTORC1 phosphorylates downstream targets including the eukaryotic initiation factor 4E (eIF4E)‐binding protein 1 (4E‐BP1) and the 70 kDa ribosomal protein S6 protein kinase (p70S6K1), leading to enhanced binding of mRNA to the ribosome for translation (Burnett et al. 1998). However, activation of mTORC1 by amino acids is blunted in muscle in conditions of anabolic resistance leading to reduced phosphorylation of 4E‐BP1 and p70S6K1 and impaired stimulation of muscle protein synthesis (Francaux et al. 2016). Thus, muscle atrophy associated with disuse is caused, in part, by an impaired response of mTORC1 to an anabolic stimulus.

In a recent study, we showed that anabolic resistance develops within 24 h of disuse induced by hindlimb immobilization in rats (Kelleher et al. 2013). In that study, a fiberglass cast was applied to one hindlimb for various periods of time, and oral administration of leucine (Leu) was used as the anabolic stimulus to overnight‐fasted animals. Within 24 h of immobilization the response of protein synthesis to the anabolic stimulus was reduced in the soleus muscle of the immobilized compared to the contralateral non‐immobilized limb and Leu‐induced activation of mTORC1 was severely blunted, effects that were maintained for at least 7 days. The impaired activation of mTORC1 by Leu was associated with upregulated expression of the mRNA encoding the mTORC1 repressor regulated in development and DNA damage responses (REDD1). However, whether or not the REDD1 protein was similarly upregulated was not assessed due to a lack of an antibody that recognizes the rat protein. Moreover, a supraphysiological concentration of Leu (1.35 g/kg) was used as the anabolic stimulus. Thus, the goal of this study was threefold. First, we wanted to determine if mTORC1 activation by a more physiologic anabolic stimulus, that is, re‐feeding, was impaired in muscle during disuse via unilateral hindlimb immobilization; second, we wanted to extend the previous study to assess whether or not REDD1 protein expression was upregulated by immobilization; and third, we wanted to determine whether or not other upstream regulators of mTORC1 activity might also be affected. We hypothesized that re‐feeding would prove inadequate to stimulate mTORC1 activity in the immobilized compared to the contralateral control limb and that the resistance of mTORC1 to activation by re‐feeding would be associated with altered expression of proteins known to modulate its function, namely increased REDD1 and DEPTOR, and decreased Sestrin1 expression.

## Materials and Methods

### Animals

Male, C57BL/6 mice (14 week; ~25 g) were obtained from Charles River Laboratories (Wilmington, MD) and were housed within the vivarium at the Pennsylvania State University College of Medicine. Mice were maintained on a 12:12 h light‐dark cycle in a temperature‐ (23°C) controlled environment. Animals were provided rodent chow (HarlanTeklad cat. #8604; Indianapolis, IN) and water ad libitum prior to experimentation. All experimental protocols were approved by the Pennsylvania State University College of Medicine Institutional Animal Care and Use Committee.

### Experimental design

Following a 1‐week acclimation to the vivarium, mice (*n* = 14) were subjected to unilateral hindlimb cast immobilization as previously described (Lang et al. 2012). Briefly, animals were anesthetized with isoflurane, and the left hindlimb was shaved. The knee was wrapped firmly with athletic tape, and a 1.5 mL microcentrifuge tube was used to immobilize the lower limb in the plantarflexed position. The distal end of the microcentrifuge tube was removed to maintain airflow to the immobilized limb. The right hindlimb served as a contralateral control. Animals were singly‐housed for the remainder of the experiment. The day before tissue harvest, mice were deprived of food beginning at 1700 h. The next morning (~0900 h), mice were randomized to remain fasted or allowed to re‐feed for 30 min prior to sacrifice (*n* = 7/group). At that time, the mice were anesthetized and the soleus muscle was removed, snap‐frozen in liquid nitrogen, and stored at −80°C until analysis.

To demonstrate the efficacy of the cast immobilization technique as a model of disuse‐induced muscle atrophy, an additional cohort of mice (*n* = 7) was unilaterally cast immobilized for a 3‐day period. After 2 days of immobilization, mice were fasted overnight and allowed to re‐feed for 30 min the following morning as described above. After the 30 min re‐feeding, mice (*n* = 3) were administered puromycin dihydrochloride (AG Scientific; San Diego, CA) in saline (0.040 *μ*mol puromycin/g body weight) via intraperitoneal injection in order to estimate rates of protein synthesis using the SUnSET method (Goodman et al. 2011). Thirty minutes post‐injection the soleus muscle was removed and snap‐frozen in liquid nitrogen before being subjected to immunoblot analysis using an anti‐puromycin antibody (Kelleher et al. 2013).

### Immunoblotting

The soleus muscle sample was Dounce homogenized in 10 volumes of buffer (10 *μ*L/mg of tissue) containing 50 mmol/L HEPES [pH 7.4], 0.10% Triton X100, 4 mmol/L EGTA [pH 8.0], 10 mmol/L EDTA [pH 8.0], 50 mmol/L Na_4_P_2_O_7_, 100 mmol/L ß‐Glycerophosphate, 25 mmol/L NaF, 5 mmol/L NaVO_4_, and 10 *μ*/mL protease inhibitor cocktail (Sigma Aldrich, cat. #P8340). Samples were then centrifuged at 10,000*g* for 10 min at 4°C, and protein in the supernatant was quantified via Bradford assay. Protein phosphorylation and total expression were determined via immunoblot as previously described (Kelleher et al. 2013; Gordon et al. 2014; Grainger et al. 2016). Immunoblot targets were separated via SDS‐PAGE on either Criterion TGX precast gels (Bio‐Rad; Hercules, CA) or 10% polyacrylamide gels (made in‐house) and transferred to PVDF membranes. Membranes were blocked in 5% milk in tris‐buffered saline with tween (TBST), and incubated overnight at 4°C with primary antibodies diluted in TBST. Antibodies against puromycin were produced in‐house and are now available commercially at Kerafast, Inc. (cat. #EQ0001; Boston, MA). Antibodies against p‐p70S6K1^Thr389^ (cat. #9205) and p‐4E‐BP1^Ser65^ (cat. #9451) were obtained from Cell Signaling (Danvers, MA); antibodies against total p70S6K1 (cat. #A300‐510A) and total 4E‐BP1 (cat. #A300‐501A) were obtained from Bethyl Laboratories (Montgomery, TX), and antibodies against REDD1 (cat. #10638‐1‐AP) and SESN1 (cat. #21668‐1‐AP) were obtained from Proteintech Group (Rosemont, IL). Following incubation in appropriate secondary antibodies (1:10,000 goat‐anti‐mouse in TBST (Bethyl cat. #A120‐101P) for puromycin; 1:2000 goat‐anti‐rabbit in TBST (Cell Signaling cat. #7074) for DEPTOR; 1:7500 goat‐anti‐rabbit in TBST (Proteintech cat. #SA00001‐2) for REDD1; and 1:10,000 goat‐anti‐rabbit in TBST (Bethyl cat. #A90‐116P) for all others) the antigen‐antibody complex was visualized by enhanced chemiluminescence using a Fluorchem M imaging system (Santa Clara, CA). A Pierce Reversible Protein Stain Kit (Thermo Scientific; Rockford, IL; cat. #24585) was used prior to the blocking step to verify equal sample loading and efficient transfer.

### Statistical analysis

Figures and statistical analyses were generated using GraphPad Prism 7.0c (La Jolla, CA). All data are presented as means ± SE. Outliers were determined and removed using the ROUT method (*Q* = 1%), with the three instances being denoted in the relevant Figure Legends. Student's *t*‐test was used to assess differences in soleus muscle mass, and two‐way ANOVA was used to determine the effect of loading and feeding status on the dependent variables. Fisher's LSD was used for post hoc analyses to determine significance for immunoblot data. Significance levels were predetermined at *P* < 0.05 for all analyses.

## Results

### Effect of immobilization on muscle mass and protein synthesis

**Figure 1:**
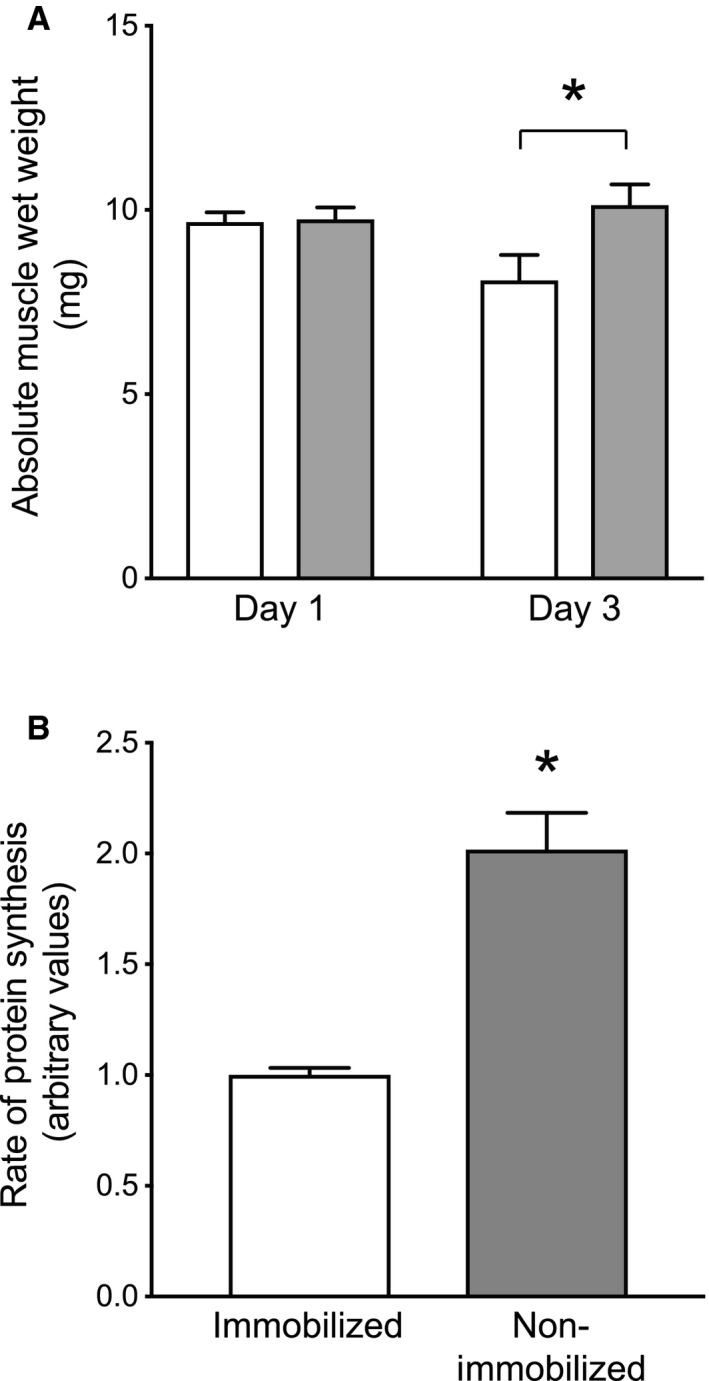
Effect of cast immobilization on soleus muscle mass and protein synthesis. (A) Absolute muscle wet weight (mg) of the soleus muscle was assessed in mice following 1 or 3 days of unilateral hindlimb immobilization, in which the left hindlimb was casted (Immobilized; open bars) and the right hindlimb served as a contralateral control (Non‐Immobilized; closed bars). The data are presented as means ± SE; *n* = 14/group for Day 1, *n* = 7/group for Day 3 values. *P < 0.05 versus Day 3 Non‐Immobilized control. (B) Protein synthesis was assessed by incorporation of puromycin into protein as described under “Materials and Methods” in mice that had been subjected to unilateral hindlimb cast immobilization, fasted overnight 2 days later, and then re‐fed for 30 min the next morning. Data presented as casted (Immobilized; open bar) versus control (Non‐Immobilized; closed bar) soleus muscle protein synthesis rates. The data are presented as means ± SE; *n* = 3/group. *P < 0.05 versus Non‐immobilized.

In agreement with the results of our previous study in rats (Kelleher et al. 2013), the mass of the mouse soleus muscle was unaltered by 24 h of hindlimb immobilization but was significantly lower in the immobilized compared to the non‐immobilized hindlimb following 72 h of immobilization (Fig. 1A). In addition, the response of muscle protein synthesis to re‐feeding was significantly blunted in the immobilized compared to the non‐immobilized limb after 3 days of immobilization (Fig. 1B).

### Effect of immobilization and fasting/re‐feeding on downstream biomarkers of mTORC1 signaling

**Figure 2:**
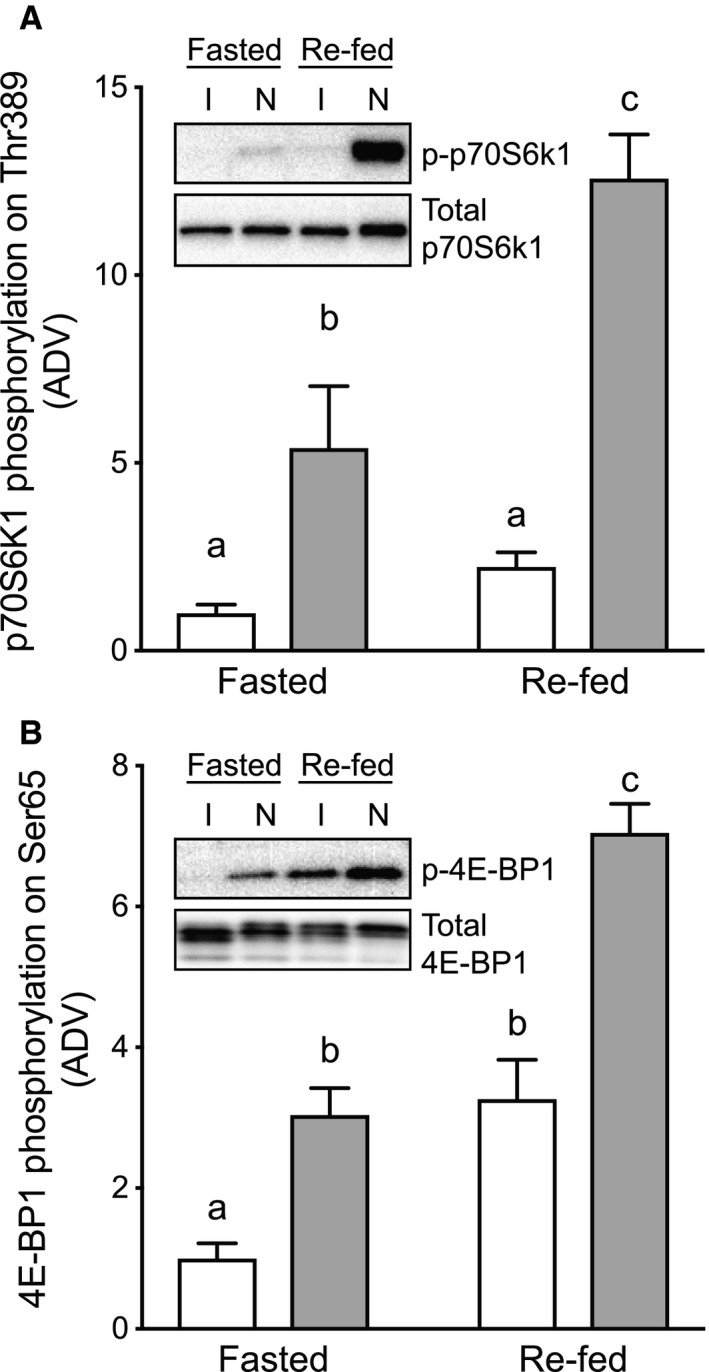
Effect of cast immobilization on phosphorylation of p70S6K1 and 4E‐BP1. Phosphorylation of (A) p70S6K1 on Thr^389^ and (B) 4E‐BP1 on Ser^65^ was assessed by Western blot analysis of muscle from mice that had been immobilized, fasted overnight, and then re‐fed for 30 min prior to sacrifice. The results are expressed as a ratio of the signal obtained for the phosphorylated protein relative to that for the total protein. Data displayed as cast immobilized (open bars) versus contralateral non‐immobilized control (closed bars). The data are presented as means ± SE of the arbitrary density values (ADV); *n* = 6–7 mice/group (1 Fasted/Non‐Immobilized outlier removed). Groups sharing a similar letter are not significantly different (*P* ≥ 0.05).

We previously demonstrated that anabolic resistance of mTORC1 to a supraphysiological bolus of Leu develops within 1 day of immobilization in rats (Kelleher et al. 2013). In agreement with those previous observations, a more physiologically relevant nutrient stimulus (i.e., re‐feeding) also failed to activate mTORC1 to the same magnitude as assessed by the phosphorylation of two of its downstream targets: p70S6K1 (Thr^389^) and 4E‐BP1 (Ser^65^) (Fig. 2A and B). Resistance of 4E‐BP1 phosphorylation to re‐feeding in the immobilized hindlimb was also apparent from changes in electrophoretic mobility during SDS‐PAGE. Thus, 4E‐BP1 mobility was noticeably increased in the immobilized compared to the contralateral control limb of fasted mice indicating reduced phosphorylation upon immobilization, and the re‐feeding‐induced decrease in mobility was blunted. Collectively, these data demonstrate that the anabolic response to a physiologically‐relevant nutrient stimulus is attenuated in immobilized skeletal muscle.

### Effect of immobilization and fasting/re‐feeding on upstream regulators of mTORC1 activity

**Figure 3:**
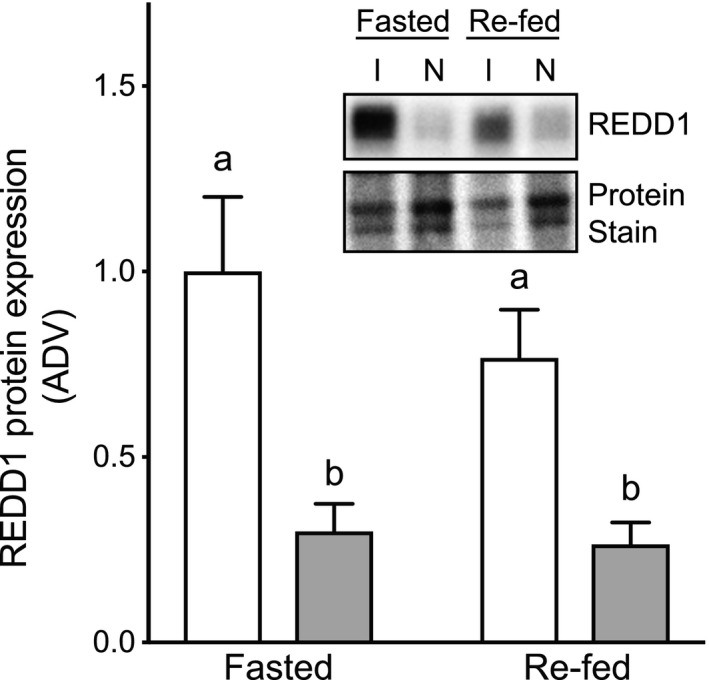
Impact of cast immobilization on REDD1 protein expression. REDD1 expression was assessed by Western blot analysis of muscle from the same mice as in Figure 2. The results are expressed as a ratio of the signal obtained for REDD1 relative to that for total protein as assessed by staining the PVDF membrane prior to blocking. Data displayed as cast immobilized (open bars) versus contralateral non‐immobilized control (closed bars). The data are presented as means ± SE of the arbitrary density values (ADV); *n* = 6–7 mice/group (1 Fasted/Non‐Immobilized outlier removed). Groups sharing a similar letter are not significantly different (*P* ≥ 0.05).

In a previous study (Kelleher et al. 2013), we showed that within 24 h of immobilization REDD1 mRNA expression was elevated in the soleus in an immobilized compared to a non‐immobilized rat hindlimb. In this study, we extend those results and show that REDD1 protein expression in the mouse soleus is also elevated in the immobilized compared to the non‐immobilized hindlimb (Fig. 3; *P* = 0.0002), but REDD1 protein content was unaffected by re‐feeding in either limb.

**Figure 4:**
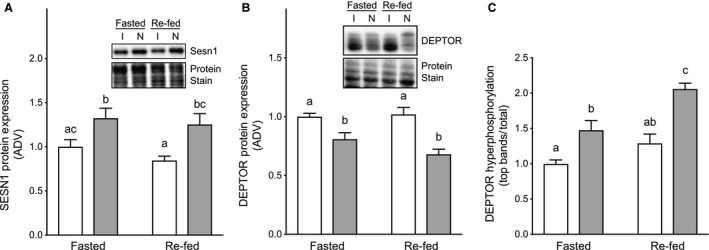
Effect of cast immobilization on Sestrin1 and DEPTOR protein expression. Expression of (A) Sestrin1 and (B) DEPTOR and (C) DEPTOR phosphorylation was assessed by Western blot analysis of muscle from the same mice as in Figures 2 and 3. In panels A and B, the results are expressed as a ratio of the signal obtained for Sestrin1 or DEPTOR relative to that for total protein as assessed by staining the PVDF membrane prior to blocking. (C) Phosphorylation of DEPTOR was assessed by changes in migration during SDS‐PAGE and expressed as the ratio of the top bands relative to all bands as depicted in the blot shown in panel B. Data displayed as cast immobilized (open bars) versus contralateral non‐immobilized control (closed bars). The data are presented as means ± SE of the arbitrary density values (ADV); *n* = 6–7 mice/group (1 Re‐fed/Non‐Immobilized outlier removed from Fig. 4B). Groups sharing a similar letter are not significantly different (*P* ≥ 0.05).

The Sestrins act as leucine “sensors” for mTORC1 because in cells lacking Sestrins, mTORC1 is resistant to leucine deprivation (Wolfson et al. 2016). Because Sestrin1 is highly enriched in skeletal muscle compared to other tissues (Peeters et al. 2003), possible immobilization‐induced changes in its expression were assessed. As shown in Figure 4A, Sestrin1 expression was repressed in the immobilized compared to the non‐immobilized hindlimb, and re‐feeding had no effect on its expression in either limb.

Because expression of the mTORC1 regulator DEPTOR is upregulated in response to many of the same stresses as REDD1, for example, DNA damage (Velasco‐Miguel et al. 1999) and glucocorticoids (Laplante et al. 2012; Singh et al. 2015), we assessed the effects of immobilization and re‐feeding on DEPTOR protein expression in the soleus. As shown in Figure 4B, expression of DEPTOR was upregulated within 24 h of immobilization. However, re‐feeding had no significant effect on expression of the protein.

DEPTOR function is regulated in part through phosphorylation, whereby phosphorylation of the protein leads to its degradation resulting in decreased DEPTOR associated with mTORC1 and consequently de‐repression of mTORC1 activity (Peterson et al. 2009). Increases in DEPTOR phosphorylation can be detected by reduced migration of the protein during SDS‐PAGE (Peterson et al. 2009). Notably, in the present study, DEPTOR phosphorylation in fasted mice was higher in the soleus of the non‐immobilized compared to the immobilized hindlimb (Fig. 4C). Re‐feeding increased DEPTOR phosphorylation in the non‐immobilized hindlimb but had no significant effect in the immobilized hindlimb. These results show that resistance of DEPTOR phosphorylation to feeding‐induced phosphorylation is a previously uncharacterized component of anabolic resistance. Overall, the data suggest that changes in mechanical load results in changes in anabolic signaling via altered expression of key mTORC1 regulators, but a 30‐min re‐feed period is insufficient in producing a nutrient‐related change in the abundance of these targets following an overnight fast.

## Discussion

This study was designed to extend our previous one (Kelleher et al. 2013) to better define the mechanisms involved in the development of anabolic resistance during disuse associated with cast immobilization. In agreement with our previous study in rats (Kelleher et al. 2013), resistance to the anabolic effect of re‐feeding occurred within 24 h of immobilization, as assessed by phosphorylation of the mTORC1 targets p70S6K1 and 4E‐BP1. Notably, the refractoriness of mTORC1 to re‐feeding was associated with upregulated expression of REDD1 protein, suggesting that increased REDD1 expression likely contributes to the blunting of the mTORC1 response to re‐feeding and oral leucine administration. This finding is consistent with the observation that, compared to wild‐type mice, mice lacking REDD1 exhibit elevated mTORC1 activity in the fasted condition (Gordon et al. 2015). Moreover, mTORC1 activity is higher both after re‐feeding fasted mice and after electrically‐induced muscle contraction in REDD1 knockout compared to wild‐type mice.

The Sestrin proteins are leucine‐binding proteins that act through a five‐subunit complex referred to as GAP activity toward Rags 2 (GATOR2) to regulate mTORC1 activity (Wolfson et al. 2016). In cells deprived of leucine, the Sestrins bind to GATOR2 and repress its ability to activate mTORC1. In contrast, in cells in leucine‐replete culture medium, the Sestrins dissociate from GATOR2 and allow it to activate mTORC1. A recent study showed that an acute bout of resistance exercise promoted increased Sestrin1, but not Sestrin2 or Sestrin3, expression in skeletal muscle of young, physically active, men (Zeng et al. 2017). In contrast, in mice subjected to an acute bout of treadmill running, Sestrin1, but not Sestrin2 protein expression was downregulated concomitant with activation of the AMP‐activated protein kinase (Crisol et al. 2018). In the present study, Sestrin1 expression was significantly reduced within 24 h of hindlimb immobilization in association with the development of anabolic resistance. Together, these studies suggest that Sestrin1 expression is directly proportional to mTORC1 activity, that is, when Sestrin1 expression is low, leucine‐induced activation of mTORC1 is impaired. In contrast, increased Sestrin1 expression would engender greater sensitivity of mTORC1 to activation by leucine. However, this conclusion appears to be inconsistent with findings in other previous studies. For example, in both liver and heart, fasting‐induced repression of mTORC1 exhibits an inverse and graded response to sequential knockout of the three Sestrins, that is, knocking out two Sestrins has a greater effect than knocking out a single one and knocking out all three has the greatest effect (Peng et al. 2014). Moreover, Sestrin expression is upregulated in response to a variety of stresses that lead to mTORC1 repression, for example, oxidative stress, hypoxia, endoplasmic reticulum stress, and DNA damage (For Review; (Parmigiani and Budanov 2016)). Based on such studies, the decrease in Sestrin1 expression observed in the immobilized hindlimb in the present study might be expected to cause mTORC1 activity to be elevated, rather than repressed. Such a conclusion is consistent with findings showing that in cells in culture, lower Sestrin2 expression is associated with greater sensitivity of mTORC1 to activation by leucine, that is, mTORC1 is fully activated at lower leucine concentrations in cells with low compared to high Sestrin 2 expression (For Review; (Wolfson and Sabatini 2017)). Although the basis for this apparent discrepancy is at this time unknown, we speculate that the drop in Sestrin1 expression observed in this study may be an adaptive mechanism, that is, a means whereby mTORC1 activity might be maintained under atrophic conditions.

A screen for proteins that bind to mTOR identified DEPTOR, the product of the *DEPDC6* gene, as a protein that associates both with mTORC1 and mTORC2 (Peterson et al. 2009). Knockdown of DEPTOR increases both mTORC1 and mTORC2 activity and, conversely, exogenous expression of the protein represses the activity of both kinases. DEPTOR expression is upregulated in cells deprived of serum, and serum re‐addition to deprived cells results in DEPTOR degradation over a 12 h time period (Peterson et al. 2009; Duan et al. 2011; Gao et al. 2011; Zhao et al. 2011). Interestingly, serum re‐addition to deprived cells rapidly (within 1 h) increases DEPTOR phosphorylation resulting in decreased affinity of the protein for mTOR as well as promoting its subsequent degradation by the proteasome.

In this study, DEPTOR protein expression was significantly increased within 24 h of hindlimb immobilization. Re‐feeding for 30 min had no statistically significant effect on DEPTOR expression, which is probably not surprising given the relatively long half‐life of the protein in cells in culture (Peterson et al. 2009). Whether or not DEPTOR abundance is decreased at longer times after re‐feeding should be addressed in future studies. During Western blot analysis of DEPTOR we noticed a distinct change in its migration during SDS‐PAGE in response to immobilization and re‐feeding. Thus, DEPTOR migrated more rapidly in samples from the immobilized compared to the contralateral non‐immobilized hindlimb regardless of feeding status, consistent with a feeding‐induced increase in phosphorylation. Moreover, although re‐feeding had no statistically significant effect on DEPTOR phosphorylation in the immobilized hindlimb, phosphorylation of the protein was significantly increased in the contralateral non‐immobilized hindlimb. Qualitatively, the pattern of change in DEPTOR phosphorylation mirrored precisely that of p70S6K1 and 4E‐BP1, an observation that is not unexpected because DEPTOR is phosphorylated in an mTORC1‐dependent manner (Peterson et al. 2009). For example, inhibition of mTORC1 with Torin1 or rapamycin not only prevents serum‐induced DEPTOR phosphorylation, but also blocks degradation of the protein (Peterson et al. 2009; Duan et al. 2011; Gao et al. 2011; Zhao et al. 2011). Thus, mTORC1‐mediated phosphorylation of DEPTOR may represent a feed‐forward mechanism fostering release of DEPTOR from mTOR allowing for both DEPTOR degradation and a further increase in mTORC1 activity.

## Conclusions

The results of this study agree with previous work showing that in rodents, anabolic resistance occurs within 24 h of disuse brought about by hindlimb immobilization. We extend those studies to show that disuse‐induced resistance of mTORC1 to nutrient stimulation is multifactorial in nature and likely manifests as a result of altered expression of several mTORC1 repressor proteins. Thus, activation of mTORC1 by re‐feeding is impaired in the muscle of an immobilized compared to a non‐immobilized hindlimb, and is associated with upregulated expression of REDD1 protein. Moreover, the expression of the mTORC1 regulatory protein DEP domain‐containing mTOR interacting protein (DEPTOR) is enhanced and expression of Sestrin1 is decreased within 24 h of immobilization, and the re‐feeding‐induced phosphorylation of DEPTOR is impaired. Overall, the results are consistent with a model in which disuse leads to downregulation of the leucine sensor, Sestrin1, and upregulated expression of mTORC1 repressor proteins including REDD1 and, based on its mRNA, REDD2 (Kelleher et al. 2013), thereby resulting in impaired nutrient‐induced activation of mTORC1. Impaired activation of mTORC1 results in a blunting in feeding‐induced DEPTOR phosphorylation leading to its stabilization and increased binding to mTORC1, thereby maintaining mTORC1 repression under anabolic conditions, for example, re‐feeding.

## Conflict of Interest

The authors have no conflicts of interest or financial ties to disclose.
